# Efficacy of portable sleep monitoring device in diagnosing central sleep apnea in patients with congestive heart failure

**DOI:** 10.3389/fneur.2022.1043413

**Published:** 2022-12-21

**Authors:** Pi-Hung Tung, Meng-Jer Hsieh, Li-Pang Chuang, Shih-Wei Lin, Kuo-Chun Hung, Cheng-Hui Lu, Wen-Chen Lee, Han-Chung Hu, Ming-Shien Wen, Ning-Hung Chen

**Affiliations:** ^1^Department of Pulmonary and Critical Care Medicine, Sleep Center, Linkou Chang Gung Memorial Hospital, Taoyuan, Taiwan; ^2^Department of Respiratory Therapy, Linkou Chang Gung Memorial Hospital, Taoyuan, Taiwan; ^3^School of Medicine, Chang Gung University, Taoyuan, Taiwan; ^4^Division of Cardiology, Department of Internal Medicine, Linkou Chang Gung Memorial Hospital, Taoyuan, Taiwan; ^5^School of Traditional Chinese Medicine, Chang Gung University, Taoyuan, Taiwan

**Keywords:** sleep apnea, central sleep apnea, heart failure, portable monitor device, polysomnography

## Abstract

**Introduction:**

Central sleep apnea (CSA) is a common and serious comorbidity mainly occurring in patients with heart failure (HF), which tends to be underdiagnosed and has not been widely studied. Overnight polysomnography (PSG) is the gold standard for diagnosing CSA; however, the time and expense of the procedure limit its applicability. Portable monitoring (PM) devices are convenient and easy to use; however, they have not been widely studied as to their effectiveness in detecting CSA in patients with HF. In the current study, we examined the diagnostic value of PM as a screening tool to identify instances of CSA among patients with HF.

**Methods:**

A total of 22 patients under stable heart failure conditions with an ejection fraction of <50% were enrolled. All patients underwent PM and overnight PSG within a narrow time frame. The measurements of the apnea–hypopnea index (AHI), hypopnea index (HI), central apnea index (CAI), and obstructive apnea index (OAI) obtained from PSG, automatic scoring, and manual scoring of PM were recorded. The results obtained from PSG and those from PM (automatic and manual scoring) were compared to assess the accuracy of PM.

**Results:**

Among the patients, CSA in 11 patients was found by PSG. The AHI measurements performed using manual scoring of PM showed a significant correlation with those performed using PSG (r = 0.69; *P* = 0.01). Nonetheless, mean AHI measurements showed statistically significant differences between PSG and automatic scoring of PM (40.0 vs. 23.7 events/hour, respectively; *P* < 0.001), as well as between automatic and manual scoring of PM (23.7 vs. 29.5 events/hour; *P* < 0.001). Central sleep apnea was detected by PM; however, the results were easily misread as obstructive apnea, particularly in automatic scoring.

**Conclusion:**

PM devices could be used to identify instances of central sleep apnea among patients with HF. The results from PM were well-correlated with standard PSG results, and manual scoring was preferable to automated scoring.

## Introduction

Congestive heart failure (CHF) is a major disorder ubiquitous in the general population (1). CHF affects more than 64 million people worldwide, and the total cost of care for HF in the United States was estimated at 43.6 billion in 2020 (2). Central sleep apnea (CSA) is indicative of a poor prognosis in patients with CHF (3); however, it tends to be underdiagnosed (4). The ability to identify instances of CSA is crucial to formulating interventions for patients with CHF.

Polysomnography (PSG) is considered the gold standard for diagnosing sleep apnea in both obstructive and central types (1, 5); however, time and labor constraints make the large-scale screening of central apnea highly impractical (6). Portable sleep monitoring (PM) is a convenient approach for screening sleep apnea, and the results for obstructive sleep apnea have been verified (7–9); however, the results for central-type sleep apnea have yet to be verified.

In this study, we assessed the efficacy of portable monitoring devices as a screening tool for central sleep apnea in patients with CHF. Also, we compared the results obtained using automated PM and manual PM scoring with those obtained using PSG.

## Materials and methods

### Patients and study protocol

**Figure 1:**
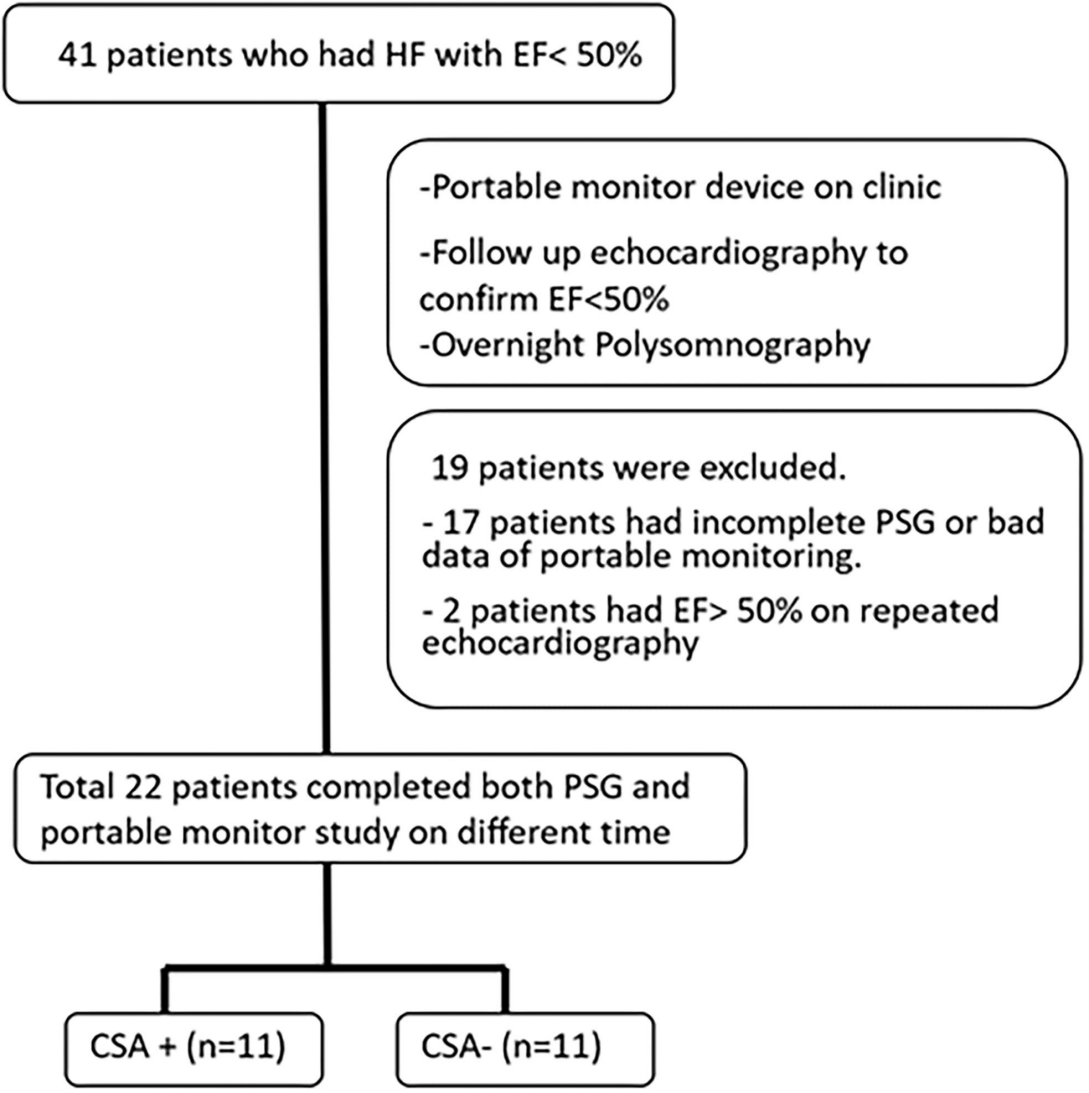
Flow diagram of the study protocol.

This study included patients who visited the cardiology clinic of the Chang Gung Memorial Hospital between June 2018 and June 2019. The inclusion criteria were adult patients (at least 20 years old), those with a left ventricular ejection fraction of <50%, and those with stable heart failure (a stable heart condition under medication control for at least 3 months prior to the study). A total of 41 patients who fit the criteria were included in the study. All patients underwent portable sleep monitoring, PSG, echocardiography, and blood tests following enrollment. PSG was performed within 1 month of testing using a portable monitoring device. A flowchart illustrating the study protocol is presented in Figure 1. Informed consent was obtained from all patients before examinations. The study complied with the guidelines of the Center for Medicare and Medicaid Service (CMS) (10). The study protocol was approved by the Institutional Review Board of the Chang Gung Memorial Hospital (IRB No. 201701305A3).

### Portable respiratory monitor

The study analysis was performed using a type 3 portable monitoring (PM) device (Medibyte, Braebon Medical Corporation, Canada), which comprised two respiratory effort bands (the chest and the abdomen), a nasal cannula pressure transducer to detect airflow, and a finger pulse oximetry sensor (oxygen saturation and heart rate) (11). Both automated scoring and manual scoring were performed, and the results were recorded. The readings obtained using the PM were manually checked by well-trained pulmonologists specializing in sleep disorders to ensure accuracy in the detection of sleep apnea.

The criteria dictating the use of the PM to score apnea and hypopneas were based on guidelines published by the American Academy of Sleep Medicine (AASM), version 2.5 (12). It should be noted that PM recordings do not allow the documentation of arousals; therefore, arousals were excluded from the definitions of central apnea and hypopnea. Apnea was defined as the cessation of inspiratory airflow for >10 s. Hypopnea was defined as a decrease in the oronasal airflow of >30% of baseline for >10 s combined with 3% oxygen desaturation. CSA was defined as the absence of a rib cage and/or abdominal excursions in the absence of airflow. OSA was defined as the absence of airflow in the presence of a rib cage and/or abdominal excursions.

### Full overnight polysomnography

Full overnight PSG was performed by a technician attendant in a sleep laboratory, and the data were analyzed in accordance with recommendations outlined in the 2014 AASM Guidelines, version 2.5 (12). PSG sensors obtained continuous recordings throughout the night to detect the body position, eye and leg movements, electroencephalography (EEG), electrooculography (EOG), electromyography (EMG), electrocardiography (ECG), and oronasal airflow by pressure monitors and thermistors, chest and abdominal effort, and pulse oximetry for confirmation. The AHI for sleep apnea was the number of apnea and hypopnea events per hour of total sleep time for polysomnography, and per hour of total recording time for portable monitoring. As mentioned earlier, the definitions of apnea, hypopnea, CSA, and OSA were the same as PM according to the 2014 AASM Guidelines.

### Echocardiography

An echocardiographic study was performed by a cardiologist using a GE Vivid Q machine (GE Healthcare, United Kingdom) to confirm ejection fractions of <50%. M-mode echocardiography and two-dimensional echocardiography were performed in standard (i.e., long-axis, short-axis, apical two-chamber, four-chamber, and subcostal) views with the patient in the supine or the left lateral position. The parameters of left ventricle (LV) dimensions and functions were measured using standard procedures, and the ejection fraction (EF) was determined using Simpson's method. In total, two patients were excluded because the EF was higher than 50%.

### Statistical analysis

Descriptive statistical analysis was performed. The data were presented as mean and standard deviation (SD), including all performance metrics. Statistical analysis was performed using Prism version 5 (GraphPad Software Inc., La Jolla, CA, USA) and SPSS Statistics version 20.0 (IBM Corporation, Armonk, NY, USA). The two groups were compared using unpaired Student's *t*-test for normal distributions, and unpaired Wilcoxon's test for non-normal distributions. A *p* < 0.05 was considered statistically significant. Bland–Altman analysis was used to study the absolute differences of AHI measurements using PM and PSG. The correlation analysis was performed using Spearman's correlation coefficients.

## Results

A total of 41 patients underwent portable monitoring, PSG, and echocardiography. Of the 41 patients, 19 patients failed to complete the study due to an inability to enter overnight PSG within 1 month, a refusal to continue due to inconvenience, an ejection fraction (EF) of >50% in follow-up echocardiographic analysis, or mortality (Figure 1). Comparisons were conducted only on the 22 patients (19 male patients; mean age = 64.1 ± 10.8 years) who completed all examinations and presented an EF of <50% (mean EF = 35.8 ± 0.9). Patient demographics are presented in Table 1.

**Table 1:** Baseline demographic characteristics of heart failure patients with or without central sleep apnea.

**Characteristics**	**All patients** **(*N* = 22)**	**With CSA** **(*N* = 11)**	**Without CSA** **(*N* = 11)**	***P*** **-value**
Age, yr	64.13 ± 10.84	64.6 ± 7.5	63.64 ± 13.8	0.83
BMI, kg/m^2^	26.46 ± 3.94	25.97 ± 4.68	26.95 ± 3.17	0.57
BNP (pg/ml)	493 ± 827.12	836 ± 1,078	150.1 ± 128.1	0.04^*^
EF, %	35.8 ± 0.89	36.0 ± 1.02	35.6 ± 0.7	0.92
**Comorbidities**				
Atrial fibrillation, *n* (%)	9 (40.9%)	5 (45.4%)	4 (36.4%)	0.66
Chronic kidney disease (eGFR), *n* (%)	84.1 ± 29.4	79.9 ± 21.55	88.2 ± 36.2	0.45
Diabetes mellitus, *n* (%)	6 (27.3%)	2 (18.2%)	4 (36.4%)	0.34
Hypertension, *n* (%)	11 (50%)	6 (54.5%)	5 (45.5%)	0.67
**NYHA Functional class**, ***n***%				0.31
I	1 (4.5%)	1 (9%)	0	
II	19 (86.3%)	10 (90.9%)	9 (81.8%)	
III	2 (9%)	1 (9.1%)	1 (9.1%)	
IV	0	0	0	
Ischemic heart disease, *n*%	10 (45.5%)	6 (54.5%)	4 (36.4%)	0.41

Data are presented as means ± SD or as a number (percentage).

* *P* < 0.05 was considered significant. BMI, body mass index; EF, ejection fraction; BNP, B-type natriuretic peptide; CSA, central sleep apnea; eGFR, estimated glomerular filtration rate; N, number.

Half of the patients (*n* = 11) experienced CSA during the overnight PSG session. The presence of CSA in patients with heart failure was associated with BNP levels exceeding those of patients without CSA (mean: 836+/−1,078 pg/ml vs. 150.1+/−128.1 pg/ml) (*P* = 0.04). No significant between-group differences were observed in terms of age, EF, etiology of heart failure, severity of heart failure (NYHA functional class), or underlying comorbidities (Table 1).

**Figure 2:**
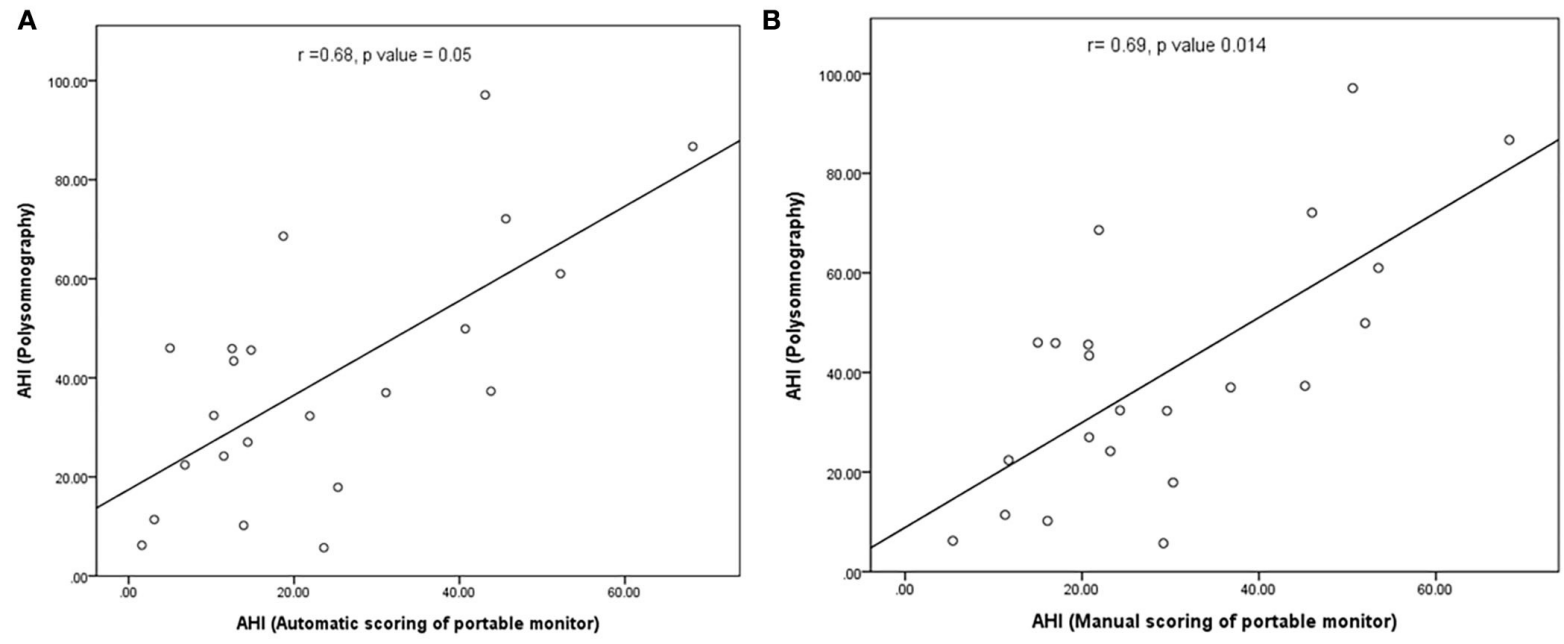
**(A)** Correlation between the number of apnea and hypopnea events in automated PM scoring and polysomnography; **(B)** correlation between the number of apnea and hypopnea in manual PM scoring and polysomnography.

**Figure 3:**
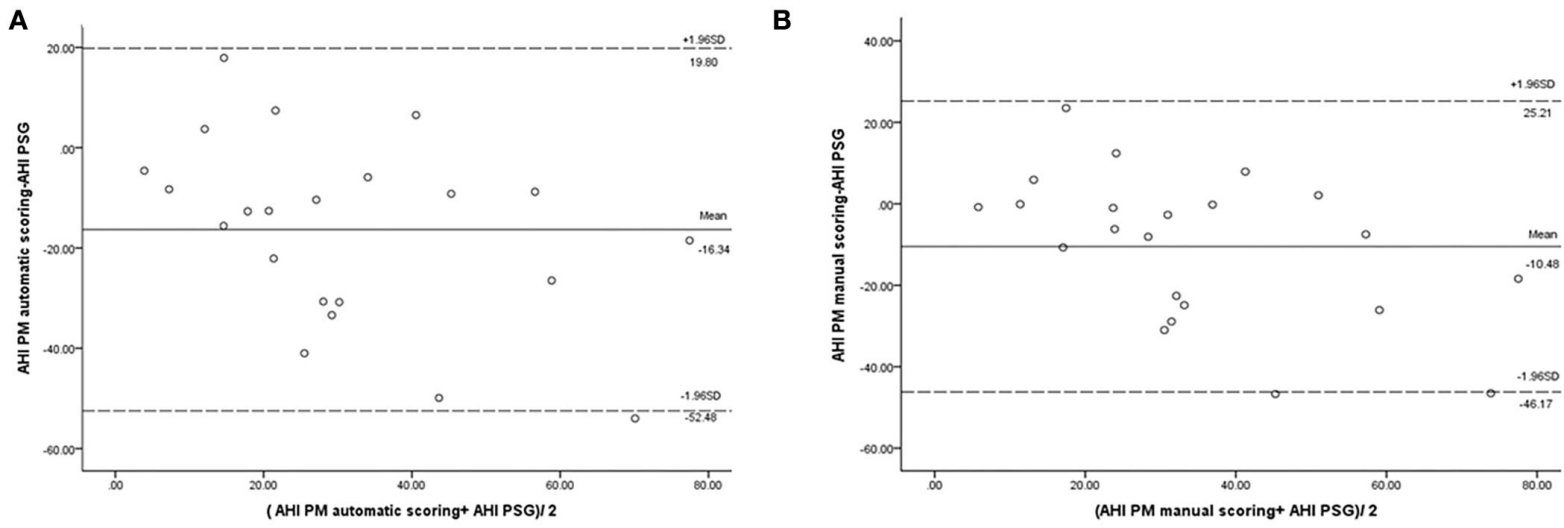
Bland–Altman plot comparing the apnea–hypopnea index: **(A)** derived from automated PM scoring and PSG; **(B)** derived from manual PM scoring and PSG.

**Figure 4:**
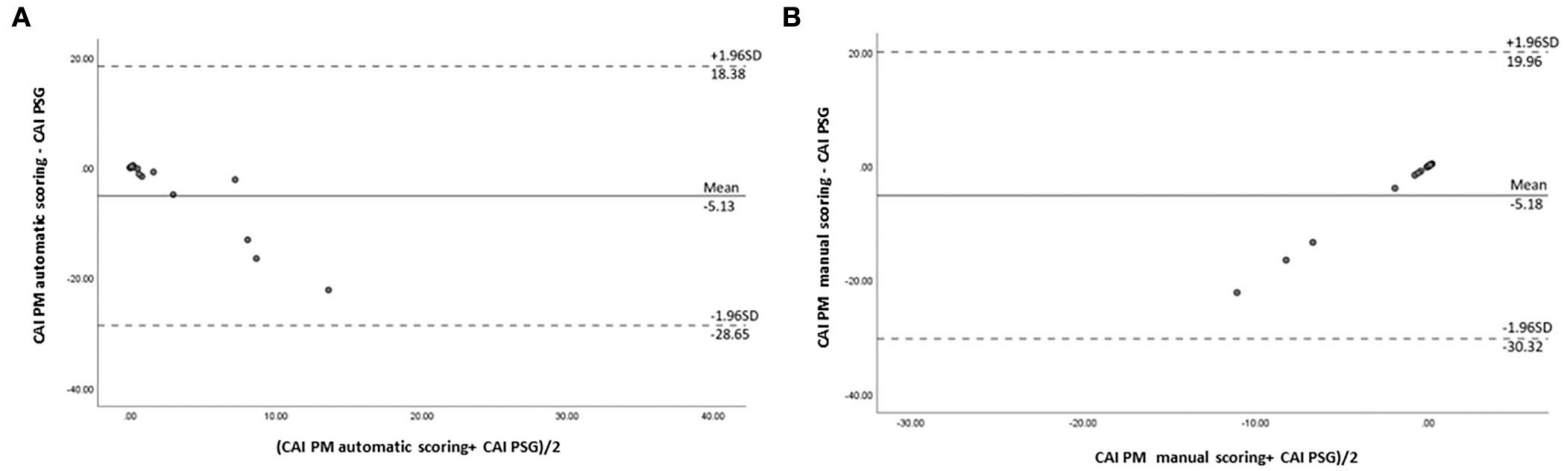
Bland–Altman plot comparing central sleep apnea indexes: **(A)** derived from automated PM scoring and PSG; **(B)** derived from manual PM scoring and PSG.

In our study, the mean sleep time recorded for all PSG tests was 242.9 ± 77.5 min, and the recorded total time for the PM tests was 378.2 ± 85.5 min. The AHI measurements obtained using the manually scored PM and PSG showed a moderately significant correlation (r = 0.69; *P* = 0.014). No correlation was observed between the automatically scored PM and PSG (r = 0.68; *P* = 0.05) (Figure 2). The Bland–Altman diagram (Figures 3, 4) illustrates absolute differences between automated PM scores and manual PM and manual PSG scores for the AHI and central sleep apnea index (CAI). The mean difference in AHI measurement obtained by automatic scoring of PM and PSG was 16.3/h, and that obtained by manual scoring of PM and PSG was 10.5/h. The mean difference in CAI measurement obtained by automatic scoring of PM and PSG was −5.1/h, and that obtained by manual scoring of PM and PSG was −5.1/h. It should be noted that 95% of the differences in the AHI ranged between −52.5 and 19.8 events per hour (automated scoring of PM and PSG) and −46.2 and 25.2 events per hour (manual scoring of PM and PSG). It should also be noted that 95% of the differences in the CAI ranged between −28.7 and 18.4 events per hour (automated scoring of PM and PSG) and −30.3 and 19.9 events per hour (manual scoring of PM and PSG).

**Figure 5:**
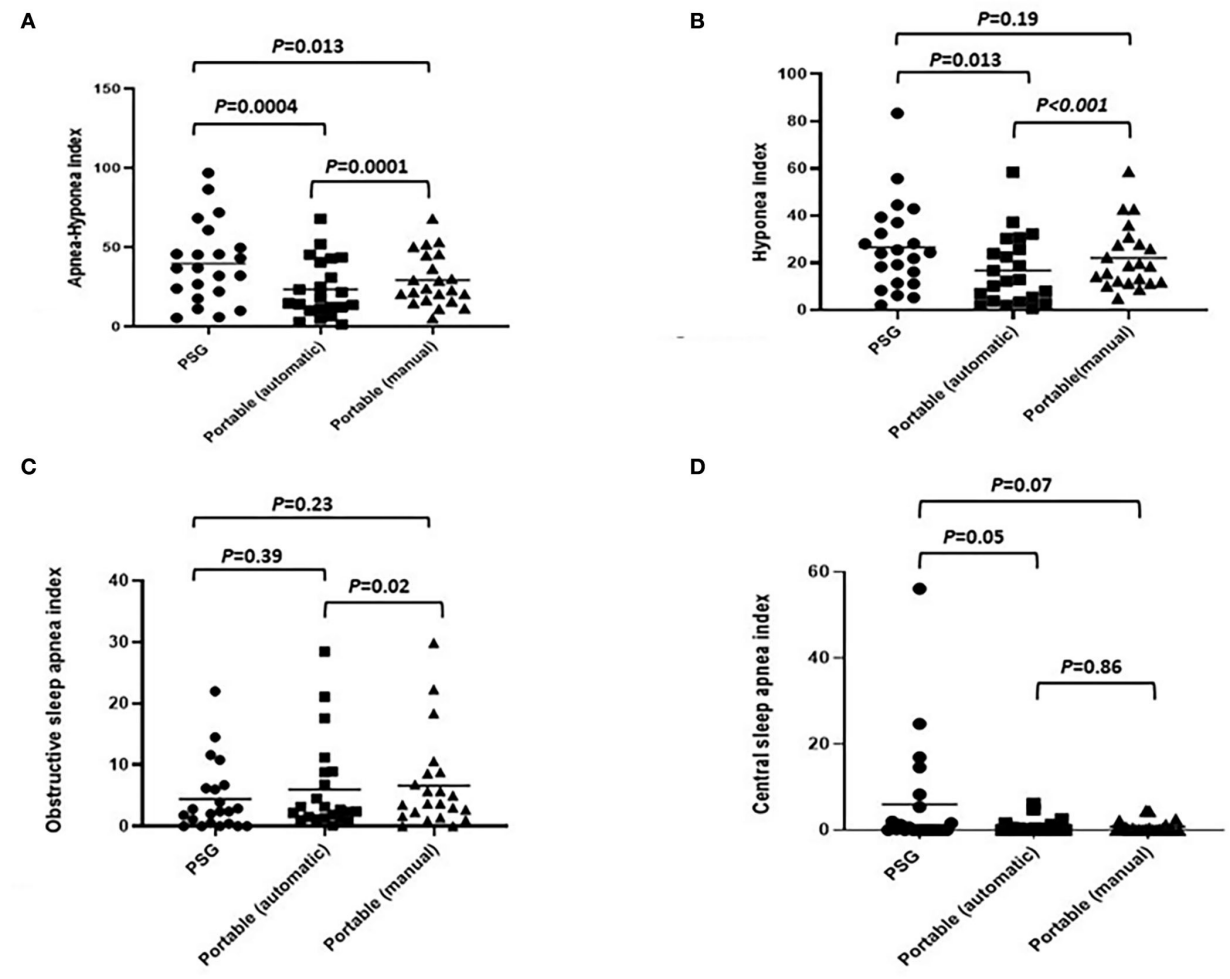
Comparison of various indexes (automated PM scoring vs. manual PM scoring vs. full overnight PSG: **(A)** apnea–hypopnea index; **(B)** hypopnea index; **(C)** obstructive apnea index; **(D)** central sleep apnea index.

The comparison of mean scores of the AHI, hypopnea index (HI), OAI, CAI, and mixed sleep apnea index (MAI) obtained by PSG with those obtained by PM scoring (automatic and manual scoring) is shown in Table 2. In the mean AHI measurement, we observed statistically significant differences between PSG and automatic scoring of PM (40.0 vs. 23.7 events/hour; *P* < 0.001), as well as between automatic and manual scoring of PM (23.7 vs. 29.5 events/hour; *P* < 0.001). In the mean scores of HI measurement, we also observed statistically significant differences between manual PSG and automated PM scores (26.6 vs. 22.1 events/hour; *P* = 0.013). The PM device reliably detected obstructive and central sleep apnea events; however, the incidence of central sleep apnea events detected by PM was lower than that detected by PSG. No statistically significant differences were observed between PSG and PM scores for the CAI or between PSG and automatic PM scores for the OAI (Figure 5).

**Table 2:** Scoring of the apnea–hypopnea index, hypopnea index, obstructive apnea index, and central sleep apnea index during full overnight session involving polysomnography or a portable monitor device using automated scoring or manual scoring.

	**Polysomnography**	**Portable monitor** **(automatic-scoring)**	**Portable monitor** **(manual-scoring)**	***P*** **-value**
Apnea-hypopnea index	40.01 ± 25.18	23.67 ± 18.01	29.53 ± 16.52	0.001^*^
Hypopnea index	26.58 ± 18.92	16.60 ± 14.74	22.06 ± 13.43	0.02^*^
Obstructive sleep apnea index	4.45 ± 5.69	6.0 ± 7.48	6.61 ± 7.66	0.29
Central sleep apnea index	5.98 ± 13.04	0.85 ± 1.62	0.8 ± 1.36	0.06
Mixed sleep apnea index	2.87 ± 4.07	NA	NA	NA

Data are presented as means ± SD.

* *P* < 0.05 was considered significant.

## Discussion

In this study, manual scoring of PM is strongly correlated with PSG in terms of the AHI, CAI, and OAI; however, the severity of PM estimate tends to be underestimated compared with PSG. The PM device was found to be effective in detecting central apnea events, but CSA events are easily misread as OSA events when measured by automatic scoring compared with when measured by manual scoring. Overall, the PM device underestimated the AHI and hypopnea events, and manual scoring was shown to be more accurate in estimating the AHI and hypopnea events than automatic scoring.

CSA is highly prevalent in patients with HF; however, most cases are undiagnosed due to a lack of typical symptoms and a lack of resources for PSG testing (6, 13). There is a pressing need for screening devices for sleep apnea that are inexpensive, easy to use, and effective. A portable monitoring device has been reported as highly reliable in detecting obstructive sleep apnea (14–16) and is widely used as a diagnostic tool in clinical practice currently. However, the diagnostic efficacy of the PM device in patients with heart failure has been questioned, and there is a lack of information on the concurrence between PM and PSG results in the detection of CSA.

The reliability of portable monitoring devices was uncertain, and researchers have yet to establish the reliability of PM devices in identifying CSA (13, 17). Aurora et al. reported that sleep apnea can be accurately identified in patients with HF using PM in an inpatient setting (13), and that a strong agreement exists between PM and PSG results for obstructive sleep apnea (77.4%) and central sleep apnea (94.3%). However, their study was conducted under well-controlled conditions with PM and PSG testing performed at the same time (at admission), which is inapplicable to real-world clinical practice. It should also be noted that the patients were surveyed at the time of admission, which means that they were suffering from acute CHF, which could be construed as selection bias. Weinreich et al. also reported that there was a high diagnostic accuracy rate for detecting central respiratory events using PM in an in-laboratory setting (17).

In the current study, PM was performed in a sleep laboratory, and each PM reading was manually checked to ensure accuracy in the detection of CSA. Our study demonstrated that PM could be used to identify central sleep apnea events; however, PM often misread central apnea events as obstructive apnea due to poor airflow signals. Thus, it appears that automated PM results should be checked manually. We observed that the scoring of PM (either automatic or manual) underestimated sleep apnea events, and that standard PSG and manual scoring were superior to automatic scoring of PM. This study demonstrates the efficacy of PM in identifying central sleep apnea. Our findings challenge previous recommendations, discouraging the use of portable sleep monitors for patients with HF or for the detection of CSA (18).

There are a few limitations to this study. First, the sample size was small due in part to a large percentage of the patients dropping out of the study. Second, PSG and PM readings could not be compared in our study since they were conducted at different time periods, unlike in previous studies in which both devices were measured at the same time. Third, we were well-aware of the fact that the Medibyte is a type 3 PM device and that there is a lack of detailed information on the efficiency of the device in detecting CSA in previous studies. Moreover, a limitation of PM is its inability to distinguish between sleep and wake periods. So, it is likely that PM underestimates the number of apnea and hypopnea incidences due to the use of total recording time, instead of total sleep time. Although PM devices are increasingly used, instead of PSG testing, in clinical practice for detecting OSA, prospective studies and large population samples are needed to verify the diagnostic accuracy of the PM device in identifying CSA.

## Conclusion

A portable monitoring device is a viable tool for the identification of central sleep apnea in patients with heart failure. The incidence of central sleep apnea in the current study was underestimated; however, PM devices provided valuable data, particularly in light of the enormous number of patients with heart failure and the difficulty in enrolling patients for PSG.

## Data availability statement

The raw data supporting the conclusions of this article will be made available by the authors, without undue reservation.

## Ethics statement

The studies involving human participants were reviewed and approved by the Institutional Review Board of Chang Gung Memorial Hospital (IRB No. 201701305A3). Written informed consent to participate in this study was provided by the patients/participants.

## Author contributions

L-PC, S-WL, N-HC, and M-SW contributed to the study conception and design. Material preparation and data collection were performed by N-HC, K-CH, M-SW, and C-HL. Data analysis was performed by P-HT, H-CH, and L-PC. The first draft of the manuscript was written by P-HT, L-PC, and N-HC. M-JH, S-WL, K-CH, C-HL, W-CL, H-CH, and M-SW commented on the previous versions of the manuscript. All authors read and approved the final manuscript.
